# Periodic knee injections of collagen tripeptide delay cartilage degeneration in rabbit experimental osteoarthritis

**DOI:** 10.1186/ar4181

**Published:** 2013-02-22

**Authors:** Takuya Naraoka, Yasuyuki Ishibashi, Eiichi Tsuda, Yuji Yamamoto, Tomomi Kusumi, Satoshi Toh

**Affiliations:** 1 Department of Orthopaedic Surgery, Hirosaki University Graduate School of Medicine, Zaifu-cho 5, Hirosaki, Aomori 036-8562, Japan; 2 Department of Pathology, Hirosaki University Graduate School of Medicine, Zaifu-cho 5, Hirosaki, Aomori 036-8562, Japan

## Abstract

**Introduction:**

Collagen peptides have been reported to possess various biological activities for various cell types. The purposes of this study were, first, to examine the therapeutic effects of collagen tripeptide (Ctp) in rabbit osteoarthritis and, second, to explore a synergetic effect with hyaluronan (HA).

**Methods:**

Osteoarthritis was induced by anterior cruciate ligament transection of the right knee in 72 Japanese white rabbits and they were divided into four groups (control, Ctp, HA and Ctp/HA). Each material was injected weekly into the knee, and knee joint samples were collected 5, 10 and 15 weeks after surgery. Macroscopic and histomorphological analyses of cartilage were conducted. Expression of type II collagen and matrix metalloproteinase-13 was also analyzed immunohistochemically. A Tukey's honestly significant difference test was used to evaluate the statistical significance of difference in the macroscopic, histological and immnohistochemical results.

**Results:**

All treatment groups exhibited slightly higher resistance to the progression of osteoarthritis than the control group macroscopically 15 weeks after surgery. Histologically, intra-articular injection of Ctp significantly reduced cartilage degradation 10 weeks after surgery, and Ctp/HA significantly reduced it 5 weeks after surgery in comparison with the control. Immunohistochemically, both Ctp-treated and Ctp/HA-treated groups had significantly increased type II collagen-positive chondrocytes at the fifth week after the surgery, although the numbers of matrix metalloproteinase-13-positive chondrocytes were not affected.

**Conclusion:**

Periodical injections of Ctp and Ctp/HA delayed progression of cartilage degeneration of early osteoarthritis induced by anterior cruciate ligament transection in rabbits. This effect appears to be exerted by promotion of type II collagen synthesis predominantly.

## Introduction

Osteoarthritis (OA) is characterized by the progressive loss of articular cartilage that leads to chronic pain and functional restrictions in affected joints. The most abundant macromolecules of the extracellular matrix of cartilage are type II collagen, aggrecan and hyaluronan (HA), and chondrocytes are the only cellular components. Under normal physiologic conditions, chondrocytes maintain an equilibrium between anabolic and catabolic activities and express various proteolytic enzymes such as aggrecanases and matrix metalloprotainases (MMPs), which mediate a very low matrix turnover responsible for cartilage remodeling. In pathologic conditions such as OA, however, production of these enzymes increases considerably, resulting in aberrant cartilage destruction [1,2]. Recent research for OA treatment has focused on finding agents aimed at reducing the catabolic factors in OA, thus slowing or reversing OA progression.

Type II collagen, one of the extracellular matrix components of cartilage [3], plays a crucial role in its tensile property, which allows formation of a fibrillar meshwork. This fibril meshwork also binds and traps proteoglycans and noncollagenous glycoproteins [4,5]. All collagens consist of three polypeptide chains, termed α chains, characterized by repeating glycine-X-Y sequences. Position × is often occupied by proline or lysine and position Y by hydroxyproline or hydroxylysine. Glycine is required at every third position to allow the close packing of α chains within the triple helix [6]. Availability of a pool of these amino acids may thus improve production of type II collagen by chondrocytes and facilitate collagen reorganization, subsequently decreasing destruction of cartilage in OA.

HA is also one of the principal components of cartilage matrix, and intra-articular injection of HA is now widely used in the treatment of OA. Exogenous HA has been shown to delay degradation of cartilage by inhibiting glycosaminoglycan release from cartilage tissue and has anti-inflammatory effects by suppressing expression of MMPs and IL-1β [7]. MMPs are involved in progressive cartilage destruction in arthritis, and MMP-13 activity has been implicated as playing a central role in cartilage degeneration in OA [8]. A previous study showed that HA inhibits IL-1β-stimulated MMP-13 production via CD44 in arthritic chondrocytes [9] and this effect of HA is also favorable to retain collagen meshwork. In addition to the effects on the cartilage, HA also suppressed MMP-13 expression in subchondral bone [10]. These effects may delay OA progression and the long-term effect of HA on OA progression has been shown in a rabbit model [11].

Collagen tripeptide (Ctp) is a highly purified, nonantigenic and low-allergenic tripeptide fraction containing Gly-Xaa-Yaa sequences [12]. Various small peptides were recently reported to possess growth factor activities [13]-Ctp exerted beneficial effects on bone fracture healing by increasing type I collagen gene expression of osteoblastic cells [14]. Furthermore, Ctp stimulated chondrocyte type II collagen gene expression in the preliminary *in vitro *study. From these findings, we hypothesized that Ctp administration within the joint would suppress the loss of cartilage matrix by increasing expression of type II collagen, subsequently preventing proteoglycan loss and promoting matrix synthesis. In addition, we hypothesized that combining Ctp and HA would bring further protective effects for knee OA progression. The objectives of this study were to investigate the protective effect of OA development by intra-articular injection of Ctp and a mixture of Ctp and HA (Ctp/HA) using an experimental model in which knee OA develops as a result of anterior cruciate ligament transection (ACLT) [15-17] in Japanese white rabbits. This study is the first to evaluate the effect of intra-articular injection of tripeptide.

## Materials and methods

### Materials

Ctp was provided by Central Research Institute, Jellice Corp. (Sendai, Japan), and HA by Seikagaku Corp. (Tokyo, Japan). Briefly, Ctp was prepared from gelatin derived from pig skin containing type I and type III collagens using a bacterial collagenase (protease-R; Roche, Basel, Schweiz) that degrades the peptide bonds of collagen at the amino-terminal end of Gly, as previously described [12]. The digest was deionized with an ion exchanger (DAION type SK; Mitsubishi Chemical, Tokyo, Japan) and then passed through a 0.2 μm filter. Further purification was performed by eliminating the endotoxins, using an ACP-0013 module (Asahi Chemical, Tokyo, Japan) and fractionation of the tripeptide fraction by reverse-phase HPLC. The tripeptide content consisting of Gly-Xaa-Yaa sequences was >96% after this purification [12]. The purity of Ctp is expressed as the content of tripeptides in the fractions, estimated from the peak integral of the absorbance at 214 nm by HPLC with a Superdex Peptide gel filtration column (Pharmacia Biotech, Uppsala, Sweden).

Solutions of 3.0 mg/ml Ctp dissolved in saline, of 3.0 mg/ml Ctp/HA in which Ctp was dissolved in HA solution (Supartz, molecular weight 800 kDa, 10 mg/ml; Seikagaku Corp.) and of HA were used. The dose of Ctp was chosen based on the local effective concentration of the previous *in vitro *preliminary study, in which type II collagen gene expressions of bovine chondrocytes were stimulated by supplementation of culture medium with 30 μg/ml Ctp (unpublished data; Y Sakai and colleagues, Jellice Corp.). We set the concentration at 3 mg/ml, 1,000 times more than the local effective concentration, because of the possibility of Ctp injected into the knee articularly being distributed throughout the body.

### Experimental animals and anterior cruciate ligament transection surgery for induction of osteoarthritis

Seventy-two mature female Japanese white rabbits were utilized in the study. Unilateral ACLT was performed under anesthesia induced by intravenous injection of 30 mg/kg sodium pentobarbital (Dainippon Sumitomo Pharma, Osaka, Japan). The anterior cruciate ligament was exposed through a medial parapatellar incision and transected at the midsubstance with a sharp blade. Complete transection of the anterior cruciate ligament was confirmed by a positive anterior drawer sign. The capsule was sutured to render it watertight, followed by skin closure. All animals were allowed normal cage activity.

All animal experiments in this study followed the Guidelines for Animal Experimentation, Hirosaki University.

### Experimental protocol for treatment

After ACLT, the rabbits were divided into four groups of 18 rabbits each: Group 1 was injected with sterile physiological normal saline as a control, Group 2 with Ctp, Group 3 with HA and Group 4 with Ctp/HA. Using a 27 G needle inserted through the lateral infrapatellar area toward the intercondylar space of the femur in each animal in a deep knee-flexed position, 0.3 ml of each reagent was administrated intra-articularly into the right knee with ACLT. The first injection was given immediately after ACLT; each of the six animals of each group were administered once weekly for 5, 10 and 15 weeks, and all animals were sacrificed by an overdose of sodium pentobarbital 1 week after the final injection was administered. The knee joints were then harvested and evaluated.

### Gross morphological examination

Gross morphological changes of the medial condyles of femur were assessed and graded as previously described [18] after application of India ink: grade 1 (score 0; intact surface), surface normal in appearance and does not retain India ink; grade 2 (score 1; minimal fibrillation), surface retains India ink as elongated specks or light-gray patches; grade 3 (score 2; overt fibrillation), areas that are velvety in appearance and retain ink as intense black patches; and grade 4 (score 3; erosion), loss of cartilage exposing the underlying bone. In a blinded manner, the assessment was conducted by two independent examiners, who were blinded to each other's findings and to the treatment group assignment of the animals. Finally, the two scores from the examiners were averaged to obtain an overall score.

### Histopathological examination

Dissected medial condyles of the femur were fixed in 10% neutral buffered formalin after gross morphological examination. Specimens were decalcified in 4% ethylemediamine tetraacetic acid solution, dehydrated with a gradient ethanol series, and embedded in paraffin blocks. Histological evaluation was performed on sagittal sections of cartilage removed from each medial condyle of the femur. Based on macroscopic observation, three serial sections of 4 μm including the most severely degenerated area of the weight-bearing lesional areas were stained with Safranin-O fast green for light microscopic examination. Histological sections were visualized using an Olympus BX41 microscope (Olympus, Tokyo, Japan) and Olympus DP2-BSW software (Olympus). One section was selected that had high quality and was representative of the three sections. Histological sections were assessed in a blinded manner by a pathologist who was unaware of the treatment group assignment of the animals, and was quantified using the histological grading system recommended by Osteoarthritis Research Society International (OARSI) [19].

### Immunohistochemical analysis

Cartilage specimens from the medial condyles of the femur were processed for immunohistochemical analysis. Three serial sections (4 μm) were stained with mouse mAb against human type II collagen (Daiichi Fine Chemical, Toyama, Japan) at a dilution of 1:100 and three serial sections (4 μm) were stained with mouse mAb against rabbit MMP-13 (CHEMICON International, Inc., Temecula, CA, USA) at a dilution of 1:25 on an automated Benchmark system (Ventana Medical Systems, Inc., Tucson, AZ, USA). In negative control sections, the primary antibody was omitted or irrelevant antibody was applied at the same concentration as the primary antibody. Image analysis was performed with multiple digital photomicrographs (Olympus) of sections taken under a high-power field (×400).

The presence of antigen in the cartilage was estimated by determining the number of chondrocytes that stained positive from the superficial zone to the upper two-thirds of hyaline cartilage. One representative section from each medial condyle of the femur was examined and scored. Before evaluation, we ensured that an intact cartilage surface for each OA specimen could be detected and used as a marker for validation of the morphometric analysis. The total number of chondrocytes and those staining positive in each section for the specific antigen were determined. The counted area was 6.76 × 10^4 ^μm^2^. Results are expressed as the percentage of chondrocytes staining positive for the antigen, with the maximum score being 100%. Each section was assessed in a blinded manner by a pathologist who was unaware of the treatment group assignment of the animals.

### Statistical analysis

All data are expressed as mean ± standard deviation. Means of groups were compared by analysis of variance and *post-hoc *test, with a Tukey's honestly significant difference test used to evaluate the statistical significance of difference in the macroscopic, histological and immnohistochemical results. *P *<0.05 was considered statistically significant.

## Results

### Gross morphological assessment

**Figure 1:**
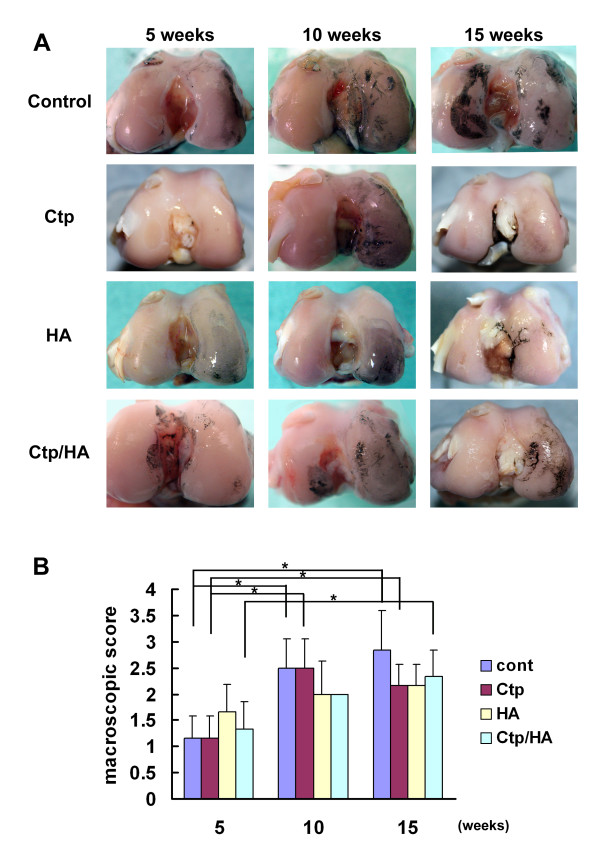
**Temporal changes in gross morphology in a rabbit osteoarthritis model**. **(A) **Macroscopic observation of articular cartilage of medial femoral condyle stained with India ink. **(B) **Macroscopic score for medial femoral condylar cartilage lesions in each group 5, 10 and 15 weeks after surgery. Ctp, collagen tripeptide; HA, hyaluronan. Data presented as mean ± standard deviation (*P <0.05).

There were no adverse effects due to injections in any rabbit, and no evidence of postoperative infection was noted. All specimens from the ACLT knees exhibited complete transection of the anterior cruciate ligament at sacrifice. All specimens exhibited changes consistent with the development of knee OA and showed mild to severe degradation of the condyle cartilage (Figure 1A). The control and Ctp injection groups exhibited significant progression of macroscopic OA score at 10 and 15 weeks compared with 5 weeks after surgery (control: 5 weeks, 1.2 ± 0.4; 10 weeks, 2.5 ± 0.5; 15 weeks, 2.8 ± 0.8; Ctp: 5 weeks, 1.7 ± 0.4; 10 weeks, 2.5 ± 0.5; 15 weeks, 2.2 ± 0.4) (*P *<0.05; Figure 1B). The score of the HA injection group also tended to increase gradually (HA: 5 weeks, 1.7 ± 0.5; 10 weeks, 2.0 ± 0.6; 15 weeks, 2.2 ± 0.5), and that of the Ctp/HA injection group was significantly increased at 15 weeks after surgery (Ctp/HA: 5 weeks, 1.3 ± 0.5; 10 weeks, 2.0 ± 0.0; 15 weeks, 2.3 ± 0.5) (*P *<0.05). However, there were no statistically significant differences in the macroscopic scores among the four groups at 5, 10 and 15 weeks (Figure 1B).

### Assessment of effect of Ctp or Ctp/HA on cartilage preservation

**Figure 2:**
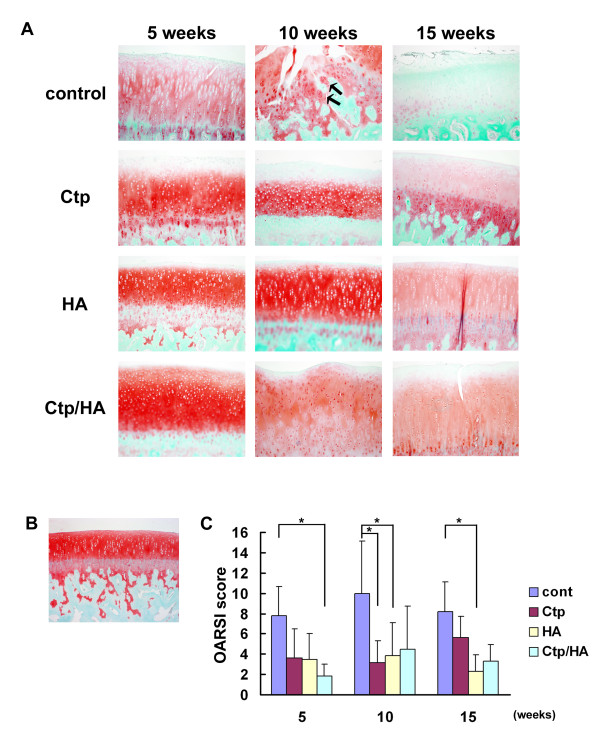
**Inhibitory effects of collagen tripeptide, hyaluronan and collagen tripeptide/hyaluronan on osteoarthritis development**. **(A) **Histologies of the medial femoral condylar cartilage stained with safranin-O. Arrow indicates cluster formation (original magnification ×100). **(B) **Appearance of safranin-O (×100) staining in the medial femoral condylar cartilage from a normal rabbit knee. **(C) **Osteoarthritis Research Society International (OARSI) **{AU Query: Confirm definition} **score for medial femoral condylar cartilage lesions in each group 5, 10 and 15 weeks after surgery. Ctp, collagen tripeptide; HA, hyaluronan. Data presented as mean ± standard deviation (*P <0.05).

In the knees of the control group, safranin-O staining was severely reduced at an early stage; and as the number of injections progressed, the gradual loss of cartilage matrix was seen (Figure 2A). In some specimens of the control group, cluster formation was recognized 5, 10 and 15 weeks after injection. Conversely, treated groups had slightly reduced safranin-O staining at five injection points, and then slowly decreased at 10 and 15 weeks after surgery (Figure 2A). Cartilage structure was maintained until a comparatively late stage. Cluster formation was also recognized in some specimens of treated groups. Histological scores for the Ctp/HA group injected for 5 weeks were significantly decreased in comparison with the control group (Figure 2B). Ten weeks after surgery, the Ctp and HA groups exhibited significantly lower scores than the control group (Figure 2B). Furthermore, the HA group maintained a lower score than the control group at 15 weeks after surgery.

### Weekly Ctp or Ctp/HA injection increases type II collagen expression during early development of osteoarthritis

**Figure 3:**
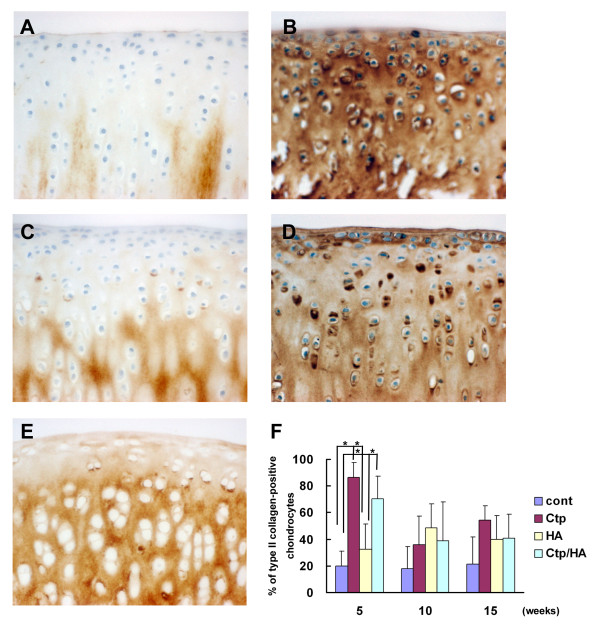
**Type II collagen expression during osteoarthritis development**. Immunohistochemical findings for type II collagen in the medial femoral condylar cartilage from **(A) **a control knee, **(B) **a collagen tripeptide (Ctp)-treated knee, **(C) **a hyaluronan (HA)-treated knee and **(D) **a Ctp/HA-treated knee 5 weeks after surgery (original magnification ×400). **(E) **Appearance of type II collagen (×400) staining in the medial femoral condylar cartilage from a normal rabbit knee. **(F) **Population of type II collagen-positive chondrocytes in medial femoral condylar cartilage of each group 5, 10 and 15 weeks after surgery. Counted area was 6.76 × 10^4 ^μm^2^. Data presented as mean ± standard deviation (*P <0.05).

The expression of type II collagen was comparable between the control group and the HA group 5 weeks after surgery (Figure 3A,C). In both groups, type II collagen was stained in a lower number of chondrocytes. In contrast, many chondrocytes expressed type II collagen in the Ctp or Ctp/HA groups at the same time points (Figure 3B,D), and the number of type II collagen-positive chondrocytes in these groups were significantly increased in comparison with the control or HA groups (Figure 3E).

### Weekly HA injection decreases MMP-13 expression during early development of osteoarthritis

**Figure 4:**
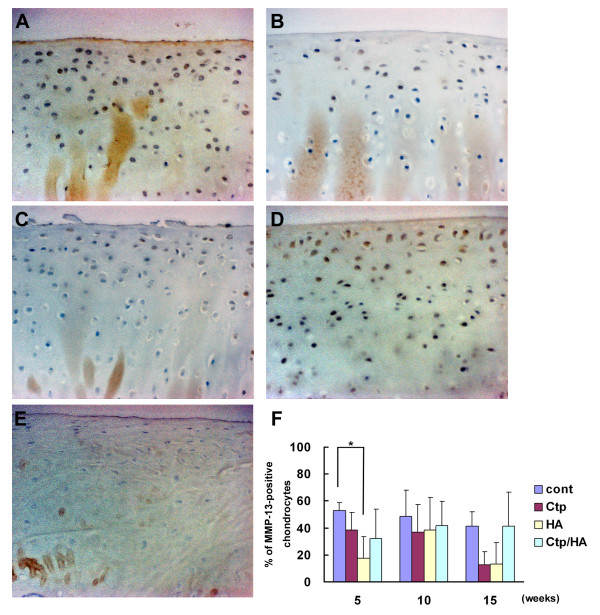
**Matrix metalloproteinase-13 expression during osteoarthritis development**. Immunohistochemical findings for matrix metalloproteinase (MMP)-13 in the medial femoral condylar cartilage from **(A) **a control knee, **(B) **a collagen tripeptide (Ctp)-treated knee, **(C) **a hyaluronan (HA)-treated knee and **(D) **a Ctp/HA-treated knee 5 weeks after surgery (original magnification ×400). **(E) **Appearance of the MMP-13 (×400) staining in the medial femoral condylar cartilage from a normal rabbit knee. **(F) **Population of MMP-13-positive chondrocytes in medial femoral condylar cartilage of each group 5, 10 and 15 weeks after surgery. Counted area was 6.76 × 10^4 ^μm^2^. Data presented as mean ± standard deviation (*P <0.05).

MMP-13 expression in chondrocytes of the HA group significantly decreased in comparison with the control group, while Ctp or Ctp/HA treatment did not have any effect on MMP-13 expression 5 weeks after surgery (Figure 4A,B,C,D,E). Ten weeks after surgery the immunostaining results of MMP-13 were not significantly different among the groups, but tended to decrease in the Ctp or HA groups 15 weeks after surgery (Figure 4E).

## Discussion

Collagen peptides have recently been reported to possess various biological activities for various cell types [13,20], and combining HA and amino acids was shown to have positive effects on the synthetic ability of fibroblasts [21]. In this study, we demonstrated that weekly injection of Ctp or Ctp/HA delayed the cartilage degeneration in a very early stage of OA progression or in the initiation of OA in an ACLT model of Japanese white rabbits.

In the control group injected with saline, significant loss of safranin-O staining and structural cartilage damage were noted 5 weeks after surgery, probably due to the decreased synthesis of type II collagen and aggrecan by chondrocytes and the subsequent increased proteolytic activity by MMPs and aggrecanases. On the contrary, a reduction of cartilage damage in the treated groups was confirmed by the OARSI scoring system. These effects on cartilage can be explained by two different mechanisms: enhancement of cartilage matrix synthesis, and inhibition of cartilage degeneration.

Our results showed that the intra-articular injection of Ctp brought a lower OARSI score 5 weeks after surgery, and significantly decreased 10 weeks after surgery in comparison with the control group. Previous studies showed that Ctp enhanced type I collagen production of human osteoblastic cells [14] and hyaluronic acid production in human dermal fibroblasts [22]. Although the mechanisms underlying the actions of Ctp for chondrocytes are not fully understood, a previous study also showed that supplementation of culture medium with tripeptide induced type II collagen synthesis predominantly by chondrocytes [13]. The results of present study showed that the number of type II collagen-positive chondrocytes was significantly increased in the Ctp treated group 5 weeks after surgery, and there was some possibility that Ctp enhanced type II collagen production of chondrocytes. Furthermore, the deposition of newly synthesized collagen necessary for cartilage reorganization may be enhanced by availability of tripeptides. Intra-articular injection of Ctp is one of the few attempts to favor the delivery of tripeptides directly to the site of the lesion, because all collagens consist of three polypeptide chains, termed α chains, characterized by repeating glycine-X-Y sequences. Sufficient supply of tripeptide is enabled to reach the chondrocytes, presumably by diffusion through the cartilage matrix via the synovial fluid and various transporter systems. Deposition of newly synthesized type II collagen necessary for cartilage anabolism may therefore be enhanced by both availability of tripeptides and activation of chondrocytes' synthetic ability, subsequently reducing cartilage degradation.

Regarding the catabolic aspect, many proteases have been shown to play major roles in the catabolism of OA cartilage. MMP-13 has been demonstrated to play a predominant role in the degeneration of type II collagen in OA cartilage [8]. In the present study, treatment with Ctp did not affect the number of MMP-13-positive cells; however, intra-articular injection of HA did significantly suppress the increase in the number of MMP-13-positive chondrocytes only 5 weeks after surgery. Previous *in vivo *study of the rabbit ACLT model showed that intra-articular injection of HA did not suppress MMP-13 mRNA expression in cartilage 10 weeks after surgery [10], and our results from the 10th and 15th weeks were consistent with the previous result. However, early intervention of HA treatment suppressed MMP-13 expression in cartilage in the early phase of our model. Previous studies have shown that HA inhibits chondrocyte MMP-13 activity through CD44 and mitogen-activated protein kinase p38 *in vitro *[12] and that MMP-13 expression in cartilage of the ACLT model was relatively higher in the early phase [10]. These results indicate that HA has chondroprotective effects in OA initiation. Moreover, our results showed that the intra-articular injection of HA significantly decreased the OARSI score 10 and 15 weeks after surgery in comparison with the control group. A previous study showed that intra-articular injection of HA suppressed MMP-13 mRNA expression in subchondral bone 10 weeks after surgery [10], and the results of our study may indicate a suppressive effect of HA on MMP-13 expression in subchondral bone.

Various scaffold material mixtures have been developed recently for tissue regeneration, exerting a synergetic effect by different biological activities [21,23]. In the present study, we performed intra-articular injection of a mixture of Ctp and HA with expectation for type II collagen synthesis and reduction of type II collagen degradation by chondrocytes. Five weeks after surgery, the Ctp/HA-treated group showed a significant increase of type II collagen-positive chondrocytes and a significant decrease of OARSI score in comparison with the control group, although the number of MMP-13-positive chondrocytes was not affected. These results suggest that the promotion of type II collagen synthesis by Ctp may contribute predominantly to the prevention of cartilage destruction. On the contrary, the intra-articular injection of Ctp/HA did not completely control MMP-13 production from articular cartilage, despite our intentions. Perhaps some kind of biological mechanism may have interfered with the Ctp/HA synergetic effect. Further study is needed to explore whether the chondroprotective effects of Ctp/HA demonstrated in the current study relate to the regulation of MMP-13 by chondrocytes.

Weekly Ctp injection suppressed progression of cartilage degeneration 10 weeks after surgery and Ctp/HA injection 5 weeks after surgery in comparison with the control group. These effects were decreased 15 weeks after surgery, however, and Ctp or Ctp/HA treatment significantly enhanced the number of type II collagen-positive cells only 5 weeks after surgery. These findings indicated that chondrocytes changed from anabolic to catabolic characteristics 10 weeks after surgery and Ctp and Ctp/HA treatment delayed only the early phase of OA. Unfortunately, these findings support our hypothesis limitedly in the initiation period of OA. The possible cause attributed to the results of the present OA model might be that the mechanical stress loaded to the articular cartilage was too strong, or that the injected concentrations of Ctp or Ctp/HA were not sufficient to neutralize the mechanical stress-induced collagen degeneration and cartilage destruction. If the synthesis of cartilage matrix could be increased further by adjusting the injection concentration of Ctp, it might be applied at the later stages of OA progression. Further examination is needed to determine the optimal dose, frequency and duration of Ctp injection therapy for the OA knee, based on *in vitro *studies and several additional *in vivo *studies.

## Conclusion

We demonstrated a novel therapeutic effect of Ctp that may be applicable to OA. Our results suggested that the intra-articular injection of Ctp and Ctp/HA seemed to be effective for the initiation period of cartilage degeneration partly by promotion of type II collagen synthesis and prevention of proteoglycan loss. We advocate that periodic intra-articular injections of Ctp have potential as a disease-modifying therapy for patients with the very early stage of OA progression.

## Abbreviations

ACLT: anterior cruciate ligament transection; Ctp: collagen tripeptide; HA: hyaluronan; HPLC: high-performance liquid chromatography; IL: interleukin; mAb: monoclonal antibody; MMP: matrix metalloproteinase; OA: osteoarthritis; OARSI: Osteoarthritis Research Society International.

## Competing interests

The authors declare that they have no competing interests.

## Authors' contributions

TN participated in the study design, acquisition of data, analysis and interpretation of data and drafting the manuscript. YI contributed to conception of this study and revised the manuscript critically for important intellectual content. ET and YY participated in study design and coordination and helped to draft the manuscript. TK carried out the immunoassays and was involved in analysis and interpretation of the data. ST gave final approval of the version to be published. All authors read and approved the final manuscript.
